# Epithelial-mesenchymal Transition (EMT) and the Effect of Atorvastatin on it in ARPE-19 cells

**DOI:** 10.1007/s12013-024-01305-w

**Published:** 2024-05-22

**Authors:** Yashavanthi Mysore, Maria Hytti, Ashik Jawahar Deen, Sofia Ranta-aho, Niina Piippo, Maija Toppila, Sirpa Loukovaara, Niina Harju, Anu Kauppinen

**Affiliations:** 1 School of Pharmacy, Faculty of Health Sciences, University of Eastern Finland, Kuopio, Finland; 2 Department of Ophthalmology, Kuopio University Hospital and School of Medicine, Faculty of Health Sciences, University of Eastern Finland, Kuopio, Finland; 3 A.I. Virtanen Institute for Molecular Sciences, University of Eastern Finland, Kuopio, Finland; 4 Department of Ophthalmology, Unit of Vitreoretinal Surgery, Helsinki University Central Hospital, and Individualized Drug Therapy Research Program, University of Helsinki, Helsinki, Finland; 5 Head and Neck Center, Ophthalmology Research Unit, Helsinki University Central Hospital, Helsinki, Finland

**Keywords:** Atorvastatin, Epithelial-mesenchymal transition, Proliferative vitreoretinopathy, Retinal detachment, Retinal pigment epithelium

## Abstract

Proliferative vitreoretinopathy (PVR) develops after an unsuccessful or complicated recovery from rhegmatogenous retinal detachment (RRD) surgery. Intraocular scar formation with the contribution of epithelial-mesenchymal transition (EMT) in RPE cells is prominent in the pathology of PVR. In the present study, the EMT process was experimentally induced in human retinal pigment epithelium (RPE; ARPE-19) cells, and the effect of atorvastatin on the process was studied. The mRNA and protein levels of mesenchymal markers actin alpha 2 (ACTA2) / alpha-smooth muscle actin (α-SMA) and fibronectin (FN), and epithelial markers occludin (OCLN) and zonula occludens-1 (ZO-1) were measured using quantitative real-time PCR (qRT-PCR) and western blot methods, respectively. In addition, α-SMA and FN were visualized using immunofluorescence staining. Cells were photographed under a phase contrast light microscope. Changes in the functionality of cells following the EMT process were studied using the IncuCyte scratch wound cell migration assay and the collagen cell invasion assay with confocal microscopy. The induction of EMT in ARPE-19 cells increased the expression of mesenchymal markers ACTA2/α-SMA and fibronectin and reduced the expression of epithelial marker OCLN both at mRNA and protein levels. The mRNA levels of ZO-1 were lower after EMT, as well. Increased levels of α-SMA and FN were confirmed by immunofluorescence staining. Atorvastatin further increased the mRNA levels of mesenchymal markers ACTA2 and FN as well as the protein levels of α-SMA and reduced the mRNA levels of epithelial markers OCLN and ZO-1 under the EMT process. EMT promoted wound closure and cell invasion into the 3D collagen matrix when compared to untreated control cells. These data present cellular changes upon the induction of the EMT process in ARPE-19 cells and the propensity of atorvastatin to complement the effect. More studies are needed to confirm the exact influence of the EMT process and atorvastatin treatment on the PVR development after RRD surgery.

## Introduction

Proliferative vitreoretinopathy (PVR)-related scar formation, the accumulation of extracellular matrix (ECM), as well as retinal pigment epithelial (RPE) cell migration and proliferation are common complications that occur after unsuccessful recovery from rhegmatogenous retinal detachment (RRD) surgery [1, 2]. The formation of epiretinal, intraretinal, and subretinal membranes is critical for the development of PVR, and the epithelial-mesenchymal transition (EMT) in RPE cells is a key factor in those pathological changes [3]. RPE cells play a major role in the maintenance of normal retinal functionality but uncontrolled proliferation and EMT in RPE cells are pathological hallmarks of PVR [4–6].

In normal physiology, EMT plays an important role in tissue development and wound healing [7]. Wound healing-related scar formation with inflammation and the growth of cellular membranes into the vitreous and retinal surfaces are typical signs of PVR [8]. RPE cells migrate to the vitreous, undergo EMT, become fibroblast-like cells, and participate in the formation of contractive epiretinal membranes [9, 10]. EMT causes both phenotypic and functional changes in RPE cells, such as disruption of tight junctions between epithelial cells, changes in cell morphology, activation of fibroblast-related [e.g., β-catenin/Wingless-integrase (Wnt) and transforming growth factor-β1 (TGF-β)] signaling pathways, and increased cell motility and proliferation rate [3, 11]. The contraction of epiretinal membranes results in the folding and wrinkling of the retina, as well as in tractional retinal detachment [8].

EMT can be experimentally induced using several growth factors, such as epidermal growth factor (EGF), fibroblast growth factor-2 (FGF-2), and transforming growth factor-β1 (TGF-β1) [12, 13]. In addition, for example, advanced glycation end products (AGE), high glucose, reduced micro-RNA (miR)-29b levels, and transcription factors, such as Snail, Slug, Twist, and forkhead box C2 (FOXC2) are capable of inducing EMT [14–19]. TGF-β is one of the most studied contributors to EMT and thereby also to the PVR formation [4, 20]. High TGF-β1 levels have been shown to participate in barrier breakdown-induced permeability of retinal endothelial cells and fibroproliferation-induced contraction of PVR-related membranes [21, 22]. EGF and FGF-2 promote EMT with proliferation via the Wnt signaling pathway, whereas TGF-β1 promotes EMT without proliferation through the Smad/Zinc finger E-box-binding homeobox 1/2 (ZEB1/2) signaling [12, 18].

We have previously shown the ability of statins (3-hydroxy-3-methylglutaryl coenzyme A reductase inhibitors; HMG-CoA reductase inhibitors) to reduce the production of pro-inflammatory interleukins (IL)-6, IL-8, and monocyte chemoattractant protein-1 (MCP-1) in human ARPE-19 cells [23]. Simvastatin prevented lipopolysaccharide (LPS) or TGF-β1-induced EMT in biliary epithelial cells and human alveolar epithelial cells, respectively, and lovastatin was capable of inhibiting EMT induced by TGF-β in porcine lens epithelial cells [24–26]. In this study, we tested the effect of atorvastatin on the EMT process in human RPE cells. We induced EMT in ARPE-19 cells using the StemXVivo EMT Inducing Media Supplement that includes recombinant proteins Wnt-5a and TGF-β1, as well as anti-E-cadherin, anti-secreted frizzled-related protein 1 (sFRP-1), and anti-dickkopf WNT signaling pathway inhibitor 1 (Dkk-1) components. Our results show reduced epithelial phenotype and increased mesenchymal properties in cells, which were further promoted by the pre-treatment of cells with atorvastatin. This is the first study on the effects of atorvastatin on EMT in human RPE cells.

## Materials and Methods

### Cell Culture

The human ARPE-19 cell line was purchased from the American Type Culture Collection (ATCC, passage 19; VA, USA). The cells were maintained on 10 cm culture plates in a humidified 5% CO_2_ atmosphere at 37 °C in Dulbecco’s modified Eagle’s medium (DMEM) and the nutrient F-12 1:1 mixture (Life Technologies, USA) containing 10% fetal bovine serum (FBS; Thermo Fisher Scientific, USA), 100 mg/ml penicillin and 100 µg/ml streptomycin (Technologies, Grand Island, NY, USA), and 2 mM L-glutamine (Lonza, Switzerland). In experiments, cells were plated at 0.7 million cells on a 10 cm plate in 7 ml culture medium containing 1X EMT-inducing media supplement (100X StemXVivo EMT Inducing Media Supplement; R&D Systems, Minneapolis, MN, USA) for 72 h. After incubation, the medium was removed and replaced with fresh pre-warmed culture medium containing 1X EMT-inducing media supplement for an additional 48 h. Medium without an EMT-inducing supplement was used as a control for the supplement. In atorvastatin studies, ARPE‐19 cells were exposed to 1.5 µM, 5 µM, or 10 µM atorvastatin for 48 h in serum-free medium, washed once with medium, and incubated with 1X StemXVivo EMT Inducing Media Supplement for 5 days. In case of western blot analyses, ARPE-19 cells were seeded on 12-well plates at the density of 200,000 cells/well for 48 h and then pre-treated with atorvastatin for 48 h, after which EMT media supplement was added for 72 h, and new EMT medium was changed for an additional 48 h. DMSO was used as a control for the atorvastatin diluent.

### Reagents and Antibodies

EMT was induced in cultured ARPE-19 cells using the StemXVivo EMT Inducing Media Supplement according to the manufacturer’s instructions. Atorvastatin (R&D systems, Sigma Aldrich, USA) was added to cultures at 1.5 µM, 5 µM, or 10 µM concentrations for 48 h in serum-free conditions and removed before the addition of 1X EMT-inducing media supplement. Mouse monoclonal antibodies anti-glyceraldehyde 3-phosphate dehydrogenase (GAPDH, Cat. ab8245, Abcam, Cambridge, UK), anti-tubulin (Cat. T5168, Sigma), anti-α-smooth muscle actin (α-SMA, Cat. MAB1420, R&D Systems), and anti-fibronectin (FN1, Cat. MAB1918, R&D Systems), as well as rabbit polyclonal anti-occludin (OCLN, Cat. 71-1500, Thermo Fisher Scientific), and rabbit monoclonal anti-N-cadherin [(D4R1H) XP #13116, Cell Signaling Technology] were used at concentrations suggested by the manufacturers (GAPDH 1:15 000, tubulin 1:10 000, fibronectin 1:1000, α-SMA 1:2500, N-cadherin 1:1000, occludin 1:2500). Horseradish peroxidase (HRP)-conjugated anti-mouse (NA931) and anti-rabbit (Novex A16104) secondary antibodies were purchased from GE Healthcare and Thermo Fisher Scientific, respectively. Cultrex^®^ rat collagen 1 (5 mg, Cat. 3440-005) was ordered from R&D Systems and diluted to 2 mg/ml concentration with 0.1% acetic acid. IncuCyte^TM^ 96-well real-time scratch wound cell migration was performed using the Cell Migration Invasion Kit (Essen BioScience Cat #4474), which includes a software application module (Cat #4400), a 96-pin IncuCyte WoundMaker tool (Cat #4563), and a 96-well ImageLock plate (Cat #4379).

### Sample Preparation

Cell lysates were collected into microtubes after the incubation of cells with 1X EMT-inducing media supplement. Cells were washed once with Dulbecco’s phosphate buffered saline (DPBS; 10 ml/plate; Lonza) before the addition of the Mammalian Protein Extraction Reagent (M-Per^®^, Thermo Scientific, 700 µl/100 mm-plate). Cell lysates were collected by scraping, centrifuged at 16,060 g for 20 min, and stored at −80 °C until analyzed. For PCR analyses, cells were collected by scraping to ice‐cold DPBS and centrifuged for 1 min at 380 *g*, +4 °C. For western blot analyses, cells from two parallel wells were pooled and 50 µl/well of M-PER (Thermo Scientific, Rockford, IL, USA) was added. Cell lysates were collected by scraping and centrifuged at 16,060 g for 20 min. Supernatants were transferred into clean microtubes and stored at −70 °C until analyzed.

### Quantitative Real-time PCR (qRT PCR) analysis

Total RNA was isolated from the lysed cells using the commercial NucleoSpin RNA/Protein extraction kit (Macherey‐Nagel, Düren, Germany). RNA purity and concentration were measured using the Nanodrop spectrophotometer (NanoDrop Technologies, Model: ND-1000) at the wavelength of 260/280 nm. cDNA was synthesized from 1000 ng of total RNA using the SuperScript™ VILO™ cDNA Synthesis Kit (Thermo Fisher Scientific). Quantitative PCR was performed using the LightCycler^®^ 480 System (Roche Molecular Systems, USA) with 384-well block according to the protocol provided with the PowerUp™/SYBR™ Green Master Mix (Applied Biosystems). The following primers (Sigma Aldrich) were used in qPCR: ACTA, forward 5′‐CTATGAGGGCTATGCCTTGCC‐3′ and reverse 5′‐GCTCAGCAGTAGTAACGAAGGA‐3′, fibronectin (FN1), forward 5′‐CGGTGGCTGTCAGTCAAAG‐3′ and reverse 5′‐AAACCTCGGCTTCCTCCATAA‐3′, Occludin transcript variant 2, forward 5′‐GACTTCAGGCAGCCTCGTTAC‐3′ and reverse 5′‐GCCAGTTGTGTAGTCTGTCTCA‐3′, ZO-1 transcript variant 1, forward 5’-ACCAGTAAGTCGTCCTGATCC-3’ and reverse 5’-TCGGCCAAATCTTCTCACTCC-3’ and two housekeeping genes, ribosomal protein large P0 (RPLP0) transcript variant 1, forward 5’-AGCCCAGAACACTGGTCTC-3’ and reverse 5’-ACTCAGGATTTCAATGGTGCC-3’ and GAPDH transcript variant 2, forward 5’-ACAACTTTGGTATCGTGGAAGG-3’ and reverse 5’-GCCATCACGCCACAGTTTC-3’. A non‐template control was included in all measurements for each gene. The mRNA expressions of ACTA2, FN1, occludin, and ZO-1 were normalized to the levels of the housekeeping genes, RPLP0 and GAPDH. Changes in the mRNA expression were calculated using the ΔΔCT method.

### Western Blot Analyses

The intracellular protein levels of fibronectin, α-SMA, N-Cadherin, occludin, GAPDH, and tubulin were determined using Western blotting. Protein levels were determined using a protocol based on the Bradford method, and proteins (20–50 µg/well) were separated in a 6% or 10% SDS-PAGE gel and transferred onto nitrocellulose membranes (GE Healthcare, Little Chalfont, UK). The membranes were blocked using 3% milk in 0.05% Tween 1X PBS for 1.5 h at room temperature. Primary antibodies were used at concentrations recommended by the manufacturer (fibronectin 1:1000, α-SMA 1:2500, N-cadherin 1:1000, occludin 1:2500), diluted in 1% milk in 0.05% Tween 1X PBS, and incubated overnight at +4 °C. Membranes were washed once with 0.3% Tween PBS for 5 min, 0.1% Tween PBS 1 × 5 min, and 0.05% Tween PBS 1 × 5 min. HRP-conjugated anti-mouse secondary antibody (NA931, GE Healthcare) was diluted 1:12 000 in 0.1% Tween/PBS and incubated on the membrane for 1 h at room temperature. HRP-conjugated anti-rabbit secondary antibody (A16104, Novex) was diluted 1:10,000 in 1% milk/0.05% Tween/PBS and incubated on the membrane for 1 h at room temperature. After secondary antibody treatments, the membranes were washed, as previously. Protein-antibody complexes were detected using the chemiluminescent method with HRP substrate (Millipore, Billerica, MA, USA) and the ImageQuant RT ECL Imager (GE Healthcare, Little Chalfont, UK) or on Super Rx medical X‐Ray film (Amersham HyperfilmTM ECL, GE Healthcare, Chicago, USA). Protein-band intensities were quantified using the ImageJ software and normalized to α‐tubulin or GAPDH values.

### Immunofluorescence staining

ARPE-19 cells were cultured on eight-well chamber slides (Ibidi GmbH, Martinsried, Germany) at the density of 6 × 10^4^ cells per well in 300 µl medium containing 1X StemXVivo EMT Inducing Media Supplement or normal culture medium. The cells were incubated for 72 h in a humidified 5% CO_2_ incubator at 37 °C, followed by a change of fresh 1X StemXVivo EMT Inducing Media Supplement or normal culture medium for an additional 48 h. After washing twice with 300 µl of DPBS per well, cells were fixed with 150 µl of 4% paraformaldehyde (PFA) at room temperature for 15 min. Then, the cells were washed three times with 300 µl of 0.1% BSA in DPBS, after which blocked and permeabilized using 300 μl of 1% BSA and 0.1% Triton X-100 in DPBS at RT for 45 min. Washing three times (á 300 µl) with 0.1% BSA in DPBS preceded the addition of mouse monoclonal α-SMA primary antibody (1:125; Cat. MAB1420, R&D Systems) or sheep polyclonal fibronectin antibody (1:500; Cat. AF1918, Bio-Techne) in 1% BSA and 0.1% Triton-X in DPBS overnight at +4 °C or for 90 min at room temperature, respectively. After the primary antibody incubation, cells were washed three times using 300 µl of 0.1% BSA in DBPS. Thereafter, 150 µl of 1:500 diluted AlexaFluor-secondary antibody anti-mouse AF488 (green) or 1:1000 diluted anti-sheep AF488 (green) antibody in DPBS with 1% BSA and 0.1% Triton X-100 was added in each well and incubated in dark at room temperature for 1 h. After that, cells were washed three times with DPBS (300 µl/well). Then, the nuclei were stained with 150 μl of 4′,6-diamidino-2-phenylindole (DAPI, Sigma) at room temperature with protection from light for 20 min, followed by washing with DPBS (3 × 300 µl). Stained cells were observed under a fluorescence microscope (Zeiss ApoTome.2 Imager M2 with the Zen Pro 2012 program).

### IncuCyte^®^ Scratch Wound Cell Migration Assay

Human RPE cells at the density of 40,000 cells/well (100 µl/well) were seeded onto 96-well ImageLock plates and grown to confluence in a 37 °C incubator, 5% CO_2_. After reaching confluency (16 h), 100 µl of culture medium without FBS was added to each well for 24 h. To measure growth-inhibited cell migration, RPE cells were pre-treated with mitomycin C (10 μg/ml, M4287, Sigma Aldrich) in starvation medium for an additional 24 h. Mitomycin C was removed by washing cells three times with DPBS (100 µl/well). Scratch wounds (700–1000 µm wide) were made on cell cultures using the WoundMaker^TM^ simultaneously in all wells according to the manufacturer’s instructions. Immediately after wounding, the culture medium was aspirated from each well, and cells were washed twice with DPBS. After washing, 100 µl/well of cell culture medium with or without the StemXVivo EMT Inducing Media Supplement was added. On day 4, the wound was inflicted again, and fresh StemXVivo EMT Inducing Media Supplement was added for 2 days. The cell plate was placed into the IncuCyte live-cell analysis, and cell migration was analyzed using a 10X objective with a phase contrast disc. The scan interval was every 2 h, and data were collected a day after the second wounding. The wound healing rate in cells containing StemXVivo EMT Inducing Media Supplement was analyzed and represented as wound width (µm/hour) and as inversed wound size (percentage of wound closed). Cells cultured in normal growth medium without the EMT supplement served as controls.

### Collagen Cell Invasion Assay

RPE cells (0.7 million/plate) were cultured on 10 cm plates in 1X StemXVivo EMT Inducing Media Supplement. After three days (day 4), cells were re-plated at 60,000 cells/well on 8-well Ibidi chambered *µ*-slides in fresh EMT-inducing media supplement. Twenty-four hours later, i.e. on day 5, the medium was discarded, and the cells were washed with DPBS/Collagen mix before casting collagen on the Ibidi slides. The cell culture at 80% confluency was coated with a 1:1 mix of 2.0 mg/ml of 3D matrix and type I collagen for an hour at 37 °C. After 1 h, cell culture medium containing EMT supplement was added on top of the solidified matrix, and the cells were allowed to invade into the matrix for 48 h. After hours, the medium was carefully removed, and cells were fixed with 4% PFA for 1.5 h at room temperature. Then cells were washed 3 × 5 min with DPBS and incubated with 200 µl 0.1 M ice-cold glycine for 30 min at room temperature. Finally, cells were washed 3 × 5 min with 200 µl DPBS and stained with 200 µl of Alexa Fluor™ 488-conjugated Phalloidin (1:40 dilution in DPBS, 150 nM, Thermo Fisher Scientific, Cat no. A12379) according to the manufacturer’s instructions for 1.5 h at room temperature. Cell nuclei were stained with DAPI (1 µM, Sigma) for 15 min at room temperature in DPBS. Ibidi slides were stored at +4 °C until analysis. Optical sections of x20 with an interval of 2 µm and a range of 55–65 slices were captured in the *Z* direction using a Zeiss LSM 700 confocal microscope and the images were stacked to get a 3D projection. Using ImageJ, the mean fluorescence of Alexa Fluor™ 488-Phalloidin and the invasion area of the cells were measured. An average of 5–10 images per group were used.

### Statistical Analyses

Statistical analyses of cell experiments were conducted using the GraphPad Prism (GraphPad Software, version 9, San Diego, CA). Differences between the groups were analyzed using the Mann–Whitney *U*-test and *P* ≤ 0.05 were considered statistically significant. Results are shown as mean ± the standard error of the mean (SEM).

## Results

### Epithelial-mesenchymal Transition in ARPE-19 Cells

**Fig. 1:**
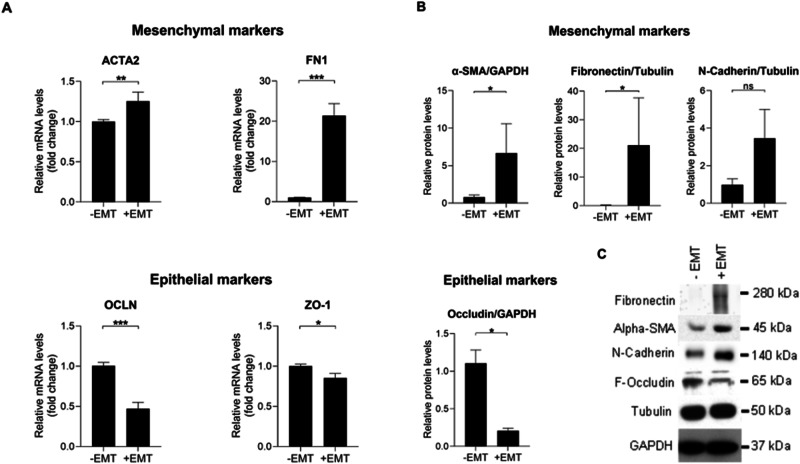
The levels of mesenchymal and epithelial markers after exposure of ARPE-19 cells to the StemXVivo EMT Media Supplement. The mRNA levels of mesenchymal (ACTA2 and FN1) and epithelial markers (OCLN and ZO-1) on day 5 (**A**). The untreated control was set to 1, and data were combined from two independent experiments with four parallel samples per group in each experiment (A; n = 8). Each sample was measured as three technical replicates in qRT-PCR. Quantitative data from the western blot analysis of fibronectin, α-SMA, N-Cadherin, and F-occludin proteins five days after the EMT induction (**B**) and a representative image from the membrane (**C**). GAPDH or tubulin served as endogenous controls. Results are combined from 3 independent experiments with 1–2 pooled samples per group in each experiment (**B**; *n* = 4, -EMT; *n* = 5, +EMT). The bars show the mean ± the standard error of the mean (SEM). *P < 0.05, **P < 0.01, ***P < 0.001, ns = not significant (Mann–Whitney *U*‐test)

**Fig. 2:**
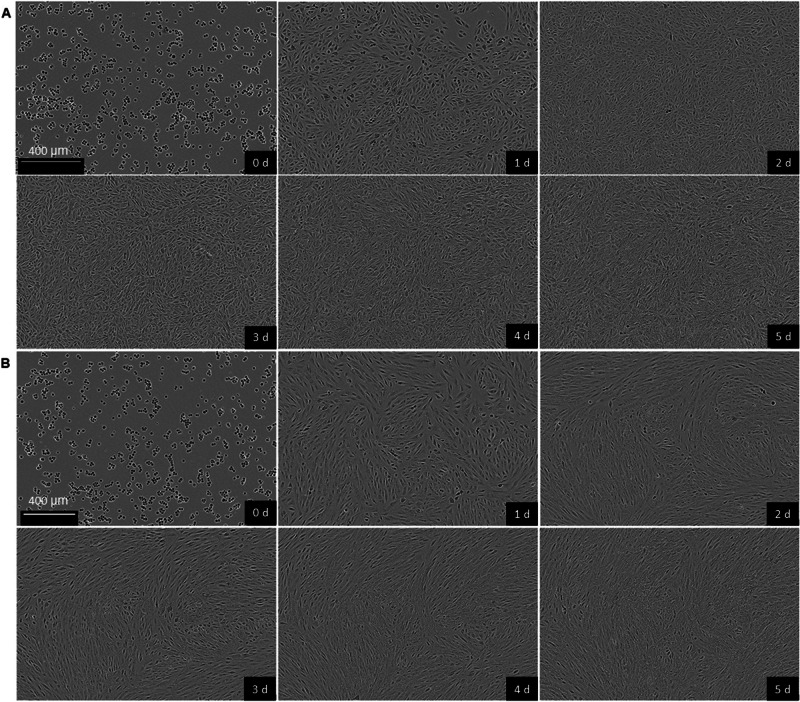
Epithelial-mesenchymal transition in ARPE-19 cells. Observations on cell morphology in untreated (**A**) and EMT-undergone (**B**) cells at different time points (0–5 days). Mesenchymal phenotype can be seen as spindle-like morphology of cells (**B**). Scale bars: 400 µm

The EMT process was verified at the molecular level by determining the mRNA levels of mesenchymal markers actin alpha 2 (ACTA2) and fibronectin 1 (FN1) as well as epithelial markers occludin (OCLN), and zonula occludens-1 (ZO-1). The levels of ACTA2 and FN1 mRNAs were significantly increased on day 5 after the induction of EMT when compared to untreated control cells (Fig. 1A). Concurrently, the levels of epithelial marker OCLN and ZO-1 mRNAs were significantly reduced (Fig. 1A). Increased expression of mesenchymal markers and decreased expression of epithelial markers were confirmed at the protein level using the western blotting technique. The increased levels of mesenchymal markers alpha-smooth muscle actin (α-SMA), encoded by the ACTA2 gene, and fibronectin, as well as the reduced levels of the epithelial marker occludin, were statistically significant (Fig. 1B, C). Only the increase in the levels of the mesenchymal marker N-cadherin did not reach statistical significance (*p* = 0.2857, Fig. 1B, C). ARPE-19 cells exposed to the EMT-inducing media supplement were observed and photographed under a phase contrast microscope. RPE cells undergone EMT were elongated, spindle-shaped, and undulatingly oriented in the cell culture compared to control cells (Fig. 2).

**Fig. 3:**
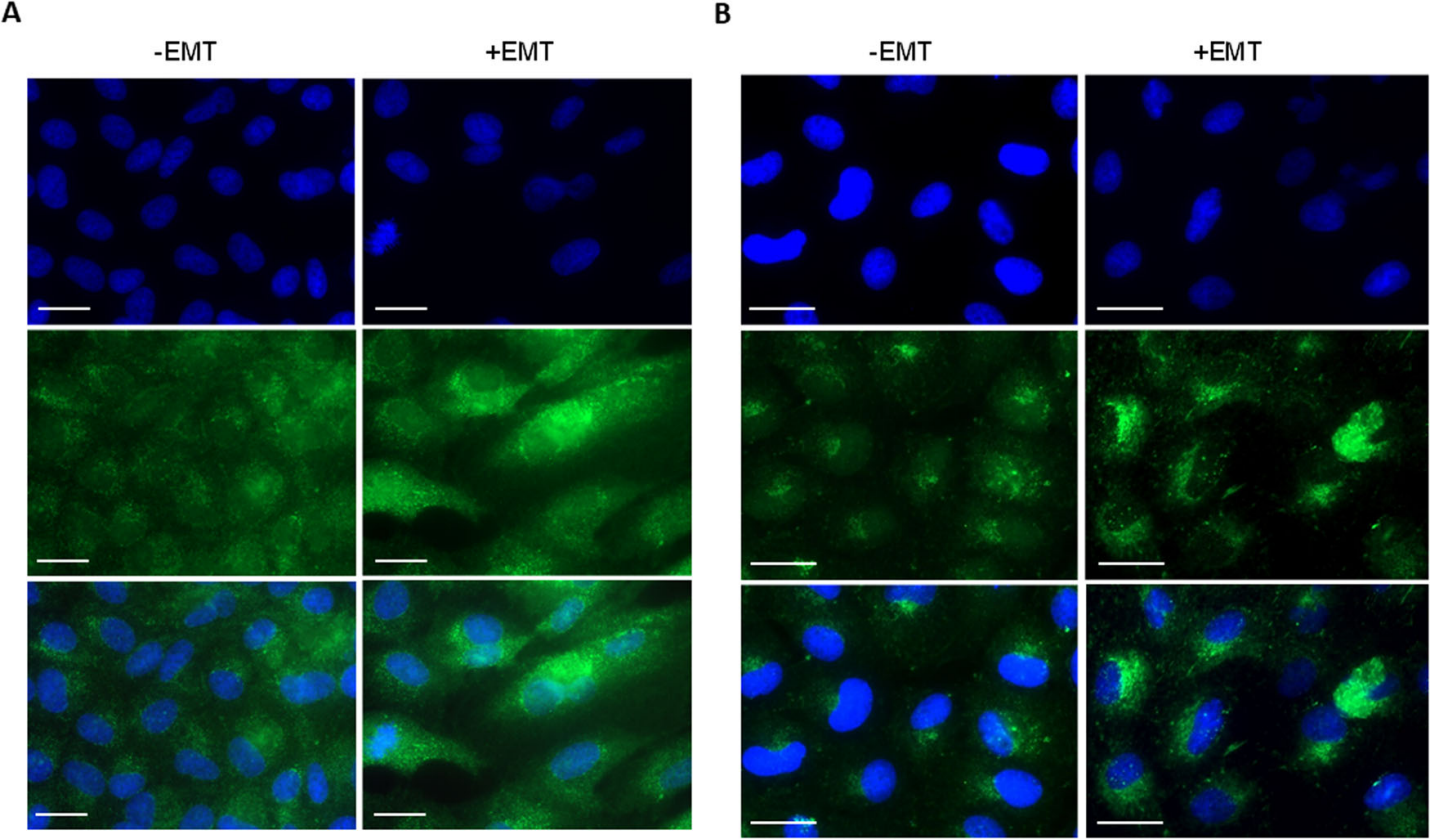
Immunofluorescence staining of mesenchymal markers in ARPE-19 cells. Representative images of α-SMA (**A**) and fibronectin (**B**). The secondary antibody for α-SMA and fibronectin were conjugated with AF488 (green), and cell nuclei were stained with DAPI (blue). Top panel; DAPI staining, middle panel; α-SMA/fibronectin, Lower panel; merged. Scale bars: 20 µm

In addition to the phase contrast microscopy, qPCR, and western blot, we demonstrated EMT by immunofluorescence staining of mesenchymal markers. EMT-inducing supplement increased the expression of α-SMA (Fig. 3A) and fibronectin (Fig. 3B) after 5 days when compared to untreated (-EMT) cells. Long, elongated, spindle-shaped morphology was seen in α-SMA-stained cells, which is considered a myofibroblast-like character related to EMT [27]. Collectively, our data show that the EMT media supplement induced epithelial to mesenchymal transition in human ARPE-19 cells.

### EMT Accelerates Wound Closure in RPE Cell Cultures

**Fig. 4:**
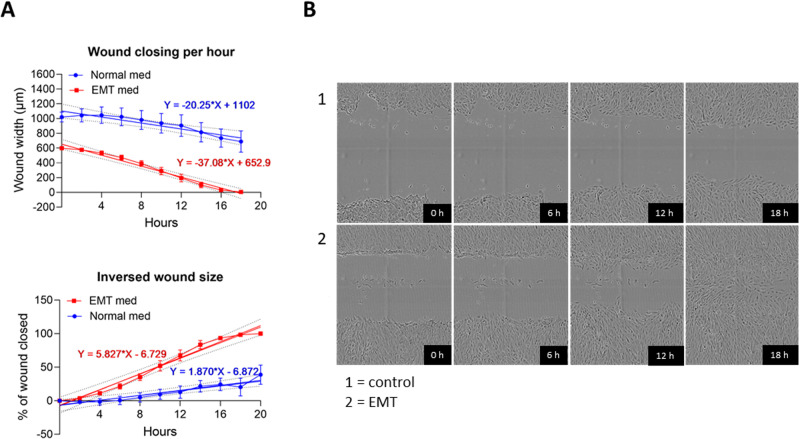
The effect of EMT induction on wound closure in ARPE-19 cell cultures. Incucyte^®^ WoundMaker induces 700 µm to 1000 µm width wound. The wound closing per hour (wound width, µm) and inversed wound size (% of the wound closed per hour) were measured with and without EMT-inducing media supplement (**A**; *n* = 5). Data are presented using simple linear regression analysis of wound areas. Representative cell images at 0 h, 6 h, 12 h, and 18 h time points after wound scratching (**B**)

To assess the cell migration of ARPE-19 cells upon StemXVivo EMT-inducing supplement treatment, we performed a scratch wound assay. Since the maximum effect of the supplement was seen after 120 h (5 days), the wound was inflicted on the cultures on day 4 using Incucyte^®^ WoundMaker. Control cells showed slower migration in comparison to cells with induced EMT at 6 h, 12 h, and 18 h time points (Fig. 4). Wound closing per hour, i.e. wound closure in terms of wound width (µm) per hour, was 1.8-fold faster in cells with EMT than in normal epithelial cells (Fig. 4A). Inversed wound size i.e. the percentage of wound closure per hour in cells with EMT-inducing media supplement showed 3.1 times faster cell migration and wound closure in comparison to control cells (Fig. 4A). At the 18 h time point, EMT cells had migrated and closed the wound, whereas untreated cells had not (Fig. 4B). Taken together, our results show that cells with EMT induction were more efficient in closing the wound in the cell culture.

### EMT-induced ARPE-19 Cells Invade Into Type 1 collagen matrix

**Fig. 5:**
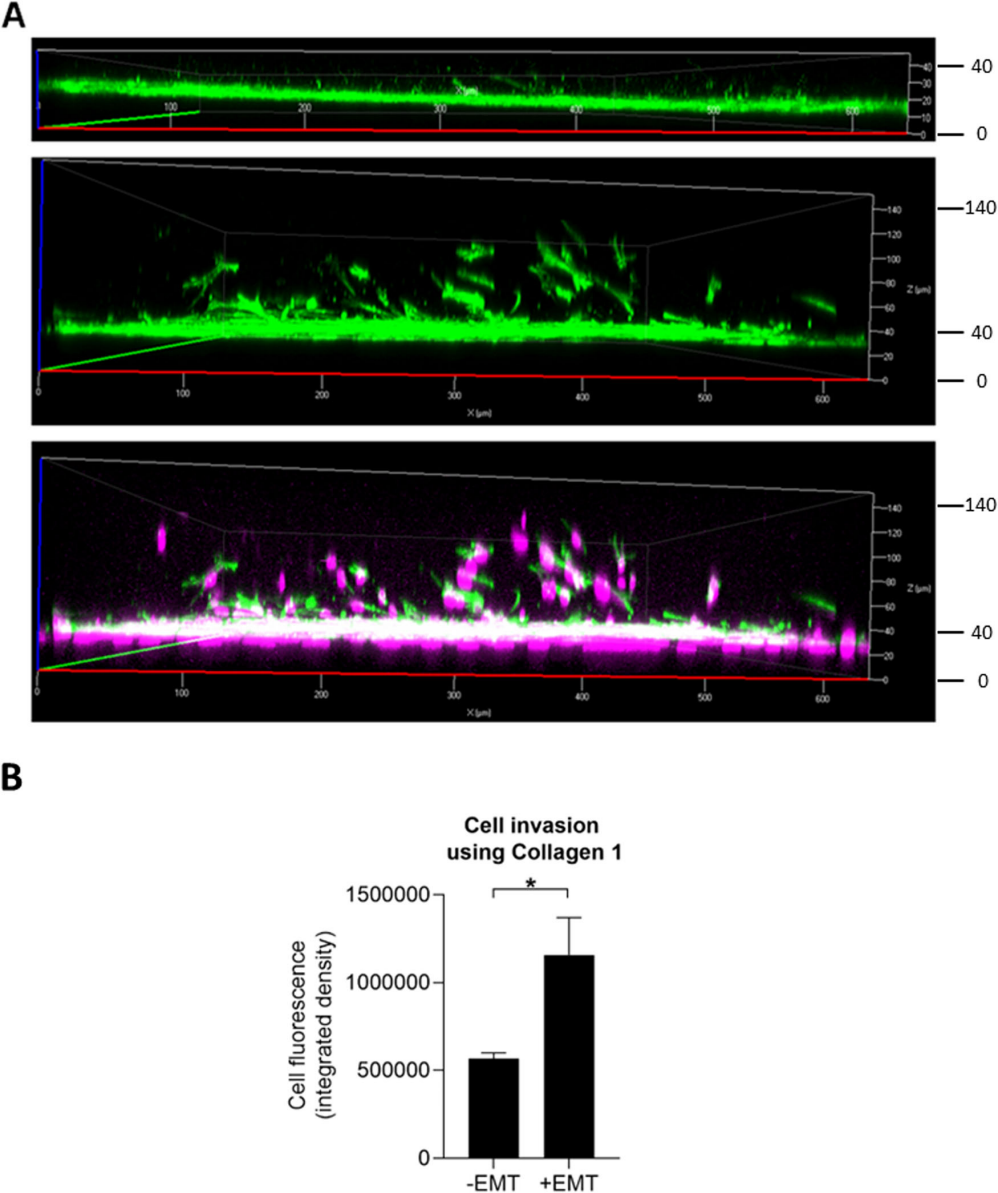
The effect of EMT-induction on cell invasion in type 1 collagen. ARPE-19 cell invasion in Type I collagen 3-dimensional invasion matrix without (top panel, single channel) or with (middle panel, single channel; lower panel, dual channels) StemXVivo EMT Inducing Media Supplement (**A**). Scale bar = µm; magenta = nuclei with DAPI staining; green = F-actin staining with the Alexa Fluor 488-phalloidin probe. Quantification of the results (**B**; *n* = 5), mean ± SEM, *p < 0.05 (Mann–Whitney U-test)

To study the invasive properties of ARPE-19 cells upon exposure to StemXVivo EMT Inducing Media Supplement, we subjected cells to a cell invasion assay using Cultrex Type 1 rat collagen. In comparison to cells cultured in a normal cell culture medium, ARPE-19 cells significantly invaded into a 3-dimensional collagen matrix when supplemented with EMT-inducing media (Fig. 5A, middle and lower panel). In addition to cell migration and invasion, filamentous actin fluorescence was more intense in EMT conditions (Fig. 5). The integrated mean cell fluorescence was significantly increased in EMT-induced cells compared to untreated cells (Fig. 5B).

### Atorvastatin Tends to Increase the Mesenchymal Marker Expression in ARPE-19 Cells

**Fig. 6:**
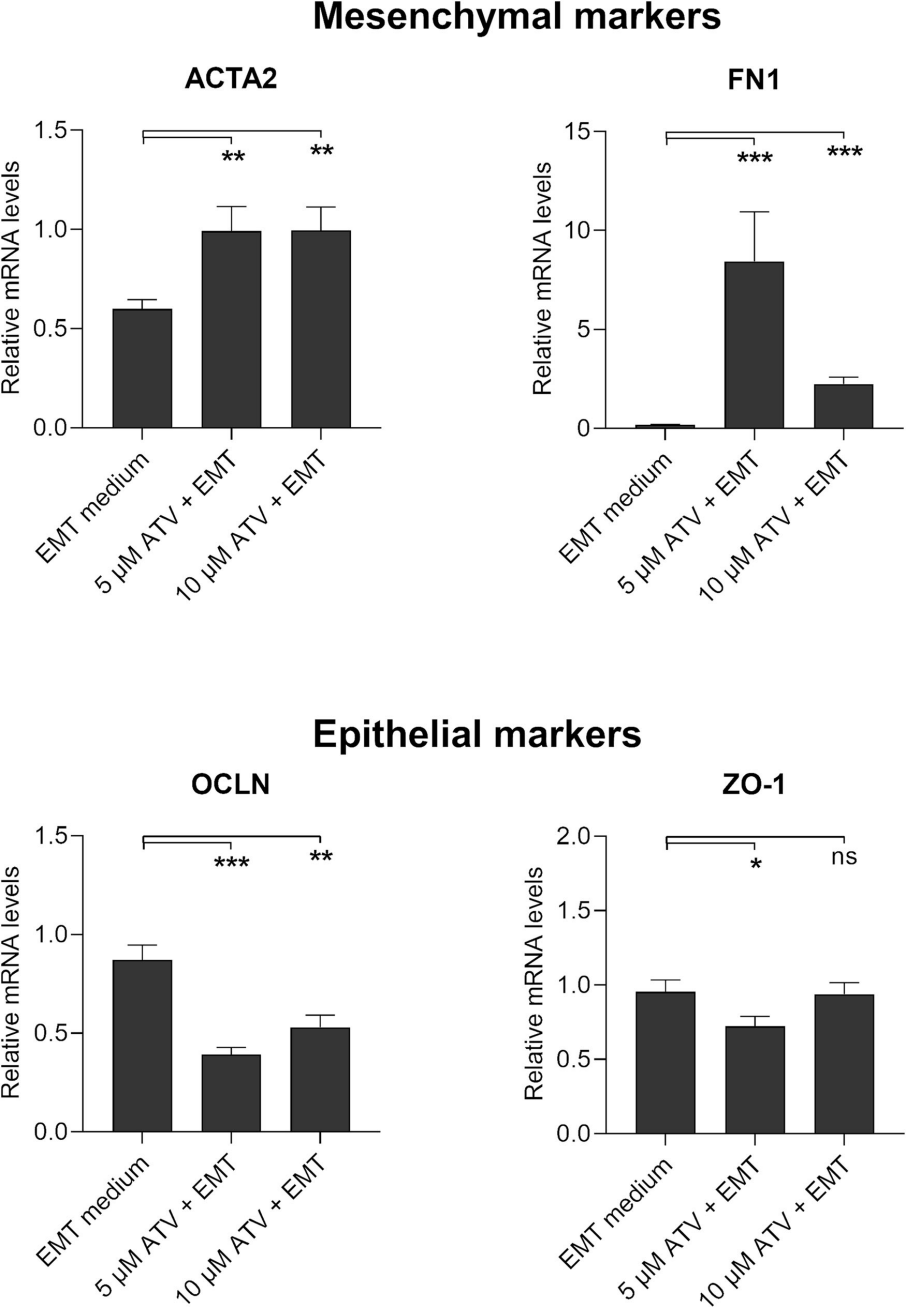
The effect of atorvastatin (ATV) on the mRNA expression of mesenchymal and epithelial markers with EMT induction in ARPE-19 cells. Data were combined from two independent experiments with four parallel samples per group. RT-qPCR samples were run in three replicates. Results are shown as mean ± SEM. *P < 0.05, **P < 0.01, ***P < 0.001; ns, not significant (Mann–Whitney *U*‐test)

**Fig. 7:**
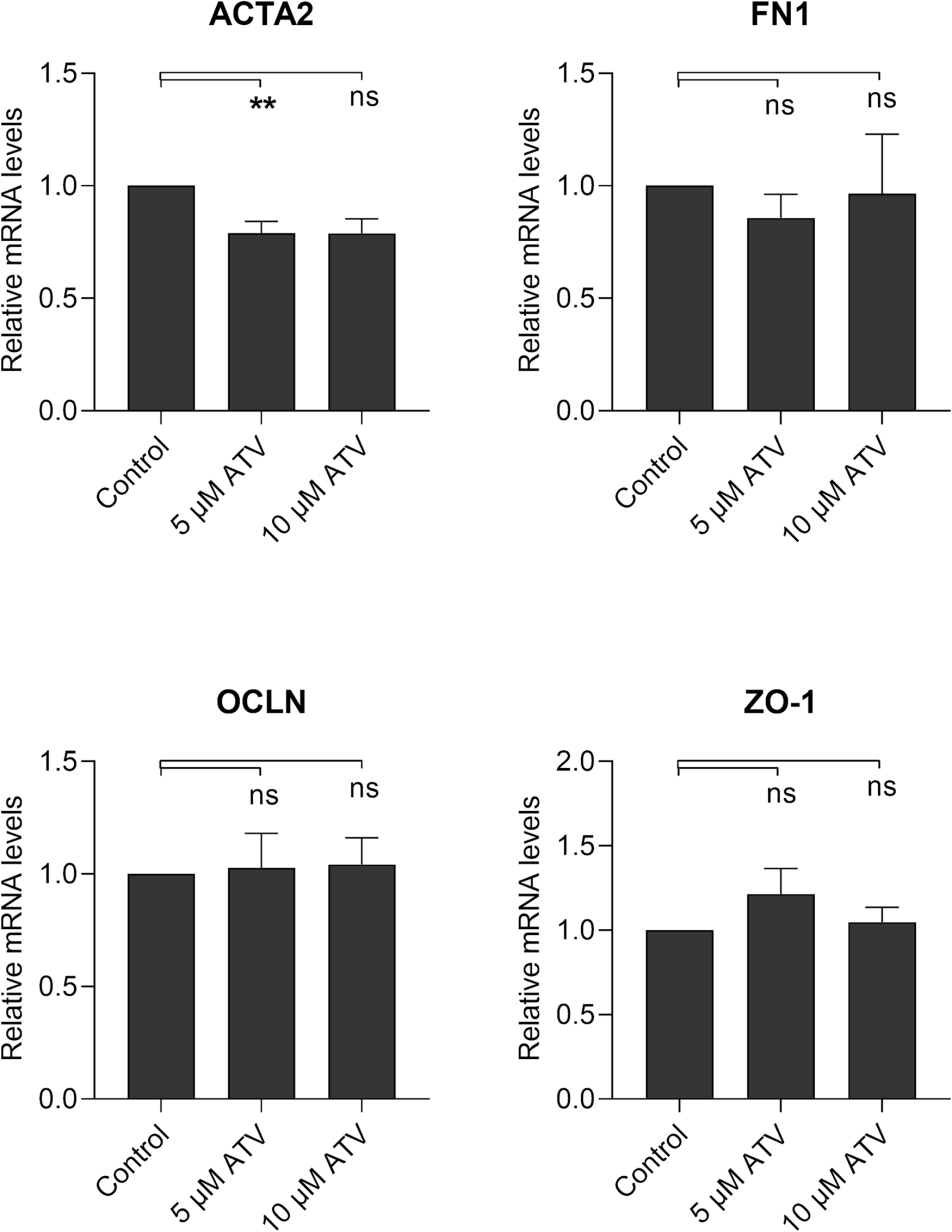
The effect of atorvastatin (ATV) on the mRNA expression of mesenchymal (ACTA2 and FN1) and epithelial (OCLN and ZO-1) markers in ARPE-19 cells without EMT induction. Data were combined from two independent experiments with four parallel samples per group in each experiment (*n* = 8). RT-qPCR samples were run in three replicates. Results are shown as mean ± SEM. **P < 0.01; ns, not significant (Mann–Whitney *U*‐test)

**Fig. 8:**
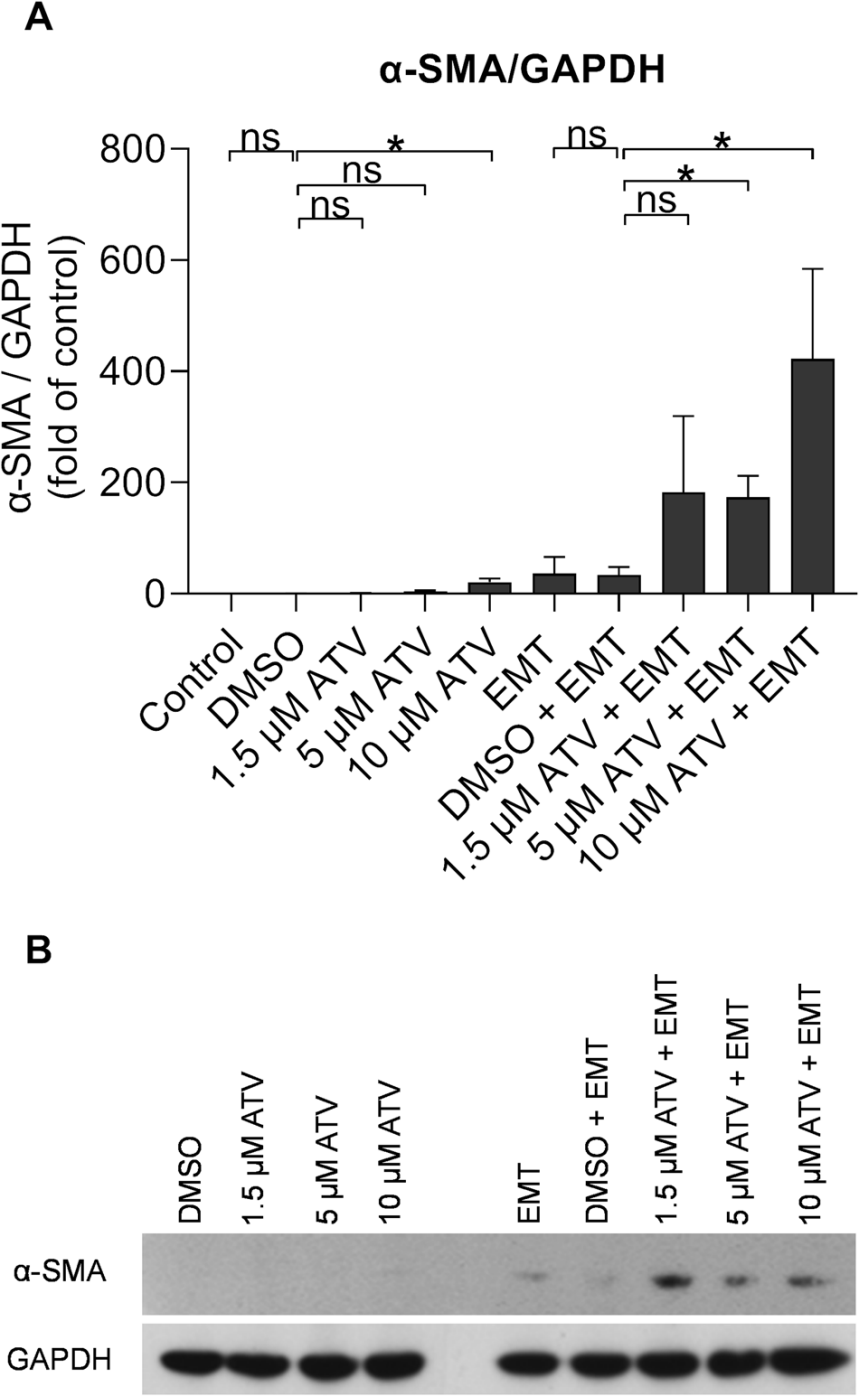
The atorvastatin (ATV) effect on the levels of α-SMA protein in ARPE-19 cells without or with EMT induction. DMSO served as dilution control to atorvastatin. The quantitation of the western blot analysis (**A**, *n* = 4) and a representative membrane image (**B**) of α-SMA at day 5. Relative protein expression was normalized to the internal protein control GAPDH. Untreated control was set to 1 (**A**), and other groups were compared to that. The bars indicate means ± SEM. Mann–Whitney *U*‐test, *P < 0.05; ns, not significant (Mann–Whitney *U*‐test)

For testing the effect of an anti-inflammatory statin on the EMT process [23], we pre-treated ARPE-19 cells with 5 µM or 10 µM atorvastatin for 48 h in serum-free medium. Atorvastatin was washed away before the addition of the EMT-inducing medium supplement. Pre-treatment with atorvastatin significantly increased the mRNA expression of the mesenchymal markers ACTA2 and FN1 (Fig. 6). Concurrently, 5 µM atorvastatin significantly reduced the mRNA expression of epithelial markers OCLN and ZO-1, and the reduction was still statistically significant with OCLN when 10 µM atorvastatin was used (Fig. 6). When atorvastatin was tested without EMT induction, it reduced mRNA levels of ACTA2 at the 5 µM concentration but had no effects on the FN1, OCLN, and ZO-1 (Fig. 7). At the protein level, atorvastatin significantly increased mesenchymal α-SMA levels at 10 µM concentration but the protein amounts remained 20 times lower in normal ARPE-19 cells in comparison to those with EMT induction (Fig. 8). These results suggest that atorvastatin can promote the expression of mesenchymal markers in ARPE-19 cells, especially upon EMT conditions.

## Discussion

EMT is a physiological process needed in embryogenesis, organ development, wound healing, and tissue regeneration [28]. It has also been associated with pathological conditions, such as the progression of cancer or the formation of scar tissue that causes tissue damage [28]. In PVR, normally post-mitotic stationary RPE cells undergo EMT, lose their cell-cell contacts, and start to proliferate and migrate to the vitreous cavity where they participate in the formation of epiretinal membranes [9, 29]. The contraction of the epiretinal membranes, in turn, causes wrinkling and (re)detachment of the retina [9]. Peng et al. (2022) showed that TGF-β-induced EMT in human RPE cells increased the levels of α-SMA and fibronectin and reduced the ZO-1 levels [30]. In the present study, EMT-inducing media supplement containing TGF-β resulted in similar outcome with increased levels of mesenchymal markers (ACTA2/α-SMA and FN1) and reduced levels of epithelial markers (occludin and ZO-1) in ARPE-19 cells. In addition, cells that undergone EMT were capable of faster wound closure and cell migration into the collagen matrix. In the eye, the proliferation and the migration of RPE cells as well as unsuccessful wound healing-related scar tissue formation are critical contributors to the development of PVR [9, 31].

Statins have shown anti-inflammatory properties in cultured ARPE-19 cells as well as potential to reduce the need for re-vitrectomy after RRD surgery in patients [23, 32]. For example, systemic statin medication was associated with 28% lower need for re-surgery of RRD [32]. In the present study, we tested the effect of atorvastatin on EMT in human ARPE-19 cells. Atorvastatin increased the levels of mesenchymal and reduced those of epithelial markers in cells subsequently exposed to EMT induction, suggesting the propensity of atorvastatin to promote EMT-related changes in RPE cells. This is in contrast to a study with human biliary epithelial cells where simvastatin alleviated rather than promoted LPS or TGF-β1-induced EMT by reducing the activation of NF-κB and the expression Toll-like receptor 4 [26] or to a study where simvastatin reduced EMT in peritoneal mesothelial cells and human proximal tubular epithelial cells [33, 34]. In cancer cells, statins have both increased and prevented EMT [25, 35–38]. Interestingly, EMT has been observed to increase the metastatic properties of cancer cells by increasing their migratory and invasion functions [35, 39]. In the development of PVR, RPE cells undergo the EMT process, start migrating, and become fibroblast or myofibroblast-like cells participating in the formation of fibrotic and contractive epiretinal membranes [3, 40]. EMT is necessary for the acute wound healing process after RRD surgery, but its uncontrolled activation can cause fibrosis [41]. Therefore, atorvastatin could be beneficial in the recovery from surgery but become detrimental if EMT remained unregulated [32]. According to the clinical data showing reduced need for re-surgery among patients with statin medication, pathological changes are not evident [32], suggesting that the EMT of RPE cells is not prolonged. A long-term study will be beneficial to see whether and how RPE cells and the scar formation are regulated by atorvastatin.

The anti-inflammatory potential of atorvastatin can be one mechanism to avoid the formation of PVR. Anti-inflammatory compounds have been proposed as potential treatment options to prevent EMT in diseases, such as biliary fibrosis or cancer [26, 42]. Metformin and heavy chain-hyaluronic acid/pentraxin 3 (HC-HA/PTX3) reduced both inflammation and EMT in RPE cells, whereas simvastatin reduced inflammation in ARPE-19 cells and EMT in a human lens epithelial cell line [23, 43–45]. Despite increased EMT in the present study, atorvastatin reduced the production of pro-inflammatory cytokines IL-6, IL-8, and MCP-1 in ARPE-19 cells [23]. The anti-inflammatory properties of atorvastatin may be related to its propensity to induce EMT since EMT has been proposed to be an attempt to control inflammation [46]. Mechanisms behind the anti-inflammatory effect of atorvastatin are also worth exploring. One limitation of the present study is that experiments have been performed using one cell line and with one statin. Due to this, the effects of other statins also deserve to be studied to reveal whether all statins behave similarly in human RPE cells. Studies performed on other cell lines including primary human RPE cells, and human epiretinal membrane samples alongside RPE cell cultures, are needed to uncover the precise effects of statins and their effects on scar tissue.

## Data Availability

Data of the current study are available from the corresponding authors upon request.
